# An *in planta* biolistic method for stable wheat transformation

**DOI:** 10.1038/s41598-017-11936-0

**Published:** 2017-09-13

**Authors:** Haruyasu Hamada, Qianyan Linghu, Yozo Nagira, Ryuji Miki, Naoaki Taoka, Ryozo Imai

**Affiliations:** 1 Hokkaido Agriculture Research Centre, National Agriculture and Food Research Organization Toyohira-ku, Sapporo, 062-8555 Japan; 2 Biotechnology Development Laboratories, KANEKA CORPORATION, Takasago, 6768688 Japan; 3 Institute of Agrobiological Sciences, National Agriculture and Food Research Organization, 2-1-2 Kannondai, Tsukuba, 305-8602 Japan

## Abstract

The currently favoured method for wheat (*Triticum aestivum* L.) transformation is inapplicable to many elite cultivars because it requires callus culture and regeneration. Here, we developed a simple, reproducible, *in planta* wheat transformation method using biolistic DNA delivery without callus culture or regeneration. Shoot apical meristems (SAMs) grown from dry imbibed seeds were exposed under a microscope and subjected to bombardment with different-sized gold particles coated with the *GFP* gene construct, introducing DNA into the L2 cell layer. Bombarded embryos were grown to mature, stably transformed T_0_ plants and integration of the *GFP* gene into the genome was determined at the fifth leaf. Use of 0.6-µm particles and 1350-psi pressure resulted in dramatically increased maximum ratios of transient GFP expression in SAMs and transgene integration in the fifth leaf. The transgene was integrated into the germ cells of 62% of transformants, and was therefore inherited in the next generation. We successfully transformed the model wheat cultivar ‘Fielder’, as well as the recalcitrant Japanese elite cultivar ‘Haruyokoi’. Our method could potentially be used to generate stable transgenic lines for a wide range of commercial wheat cultivars.

## Introduction

Wheat (*Triticum aestivum* L.) is a major staple crop that is cultivated worldwide. Difficulty in transformation has meant that the application of genetic engineering in wheat has lagged behind that of other crops, such as rice and maize. The first successful transformation of wheat was reported using particle bombardment of embryogenic callus^1^. Subsequently, an *Agrobacterium*-mediated transformation method using immature, embryo-derived, regenerable callus was developed^2–4^. To improve the efficiency of culture-based transformation, several model cultivars have been modified under various experimental conditions^5–8^. However, the application of these methods is limited to those cultivars with characteristics suitable for culture and regeneration. Many elite commercial cultivars lack this property, and are thus difficult to transform. In addition, culture-based transformation methods are generally time-consuming and suffer from somatic variations.

To avoid the problems associated with tissue culture and regeneration steps in transformation, alternative *in planta* methods, which introduce transgenes directly into intact plant tissue, have been developed in several plant species. In *Arabidopsis thaliana*, the *Agrobacterium*-mediated floral-dip method is most widely used to transform immature florets^9,10^, targeting ovule cells^10^. *In planta* transformation methods have been reported in other plant species, such as *Medicago truncatula* ^11^, *Solanum lycopersicum* and some cereals^12–15^, but these are not used as standard protocols as they are often inefficient and irreproducible.

Here we report a novel *in planta* wheat transformation method using biolistic particle delivery. *In planta* particle bombardment (iPB) utilises the meristematic tissues of mature embryos and does not require embryogenic callus culture, regeneration, or antibiotic selection. We show that this method can produce stably transformed transgenic wheat plants, not only in an experimental cultivar (‘Fielder’), but also in a commercial elite cultivar (‘Haruyokoi’).

## Results

### Optimization of bombardment conditions for *in planta* transformation

**Figure 1:**
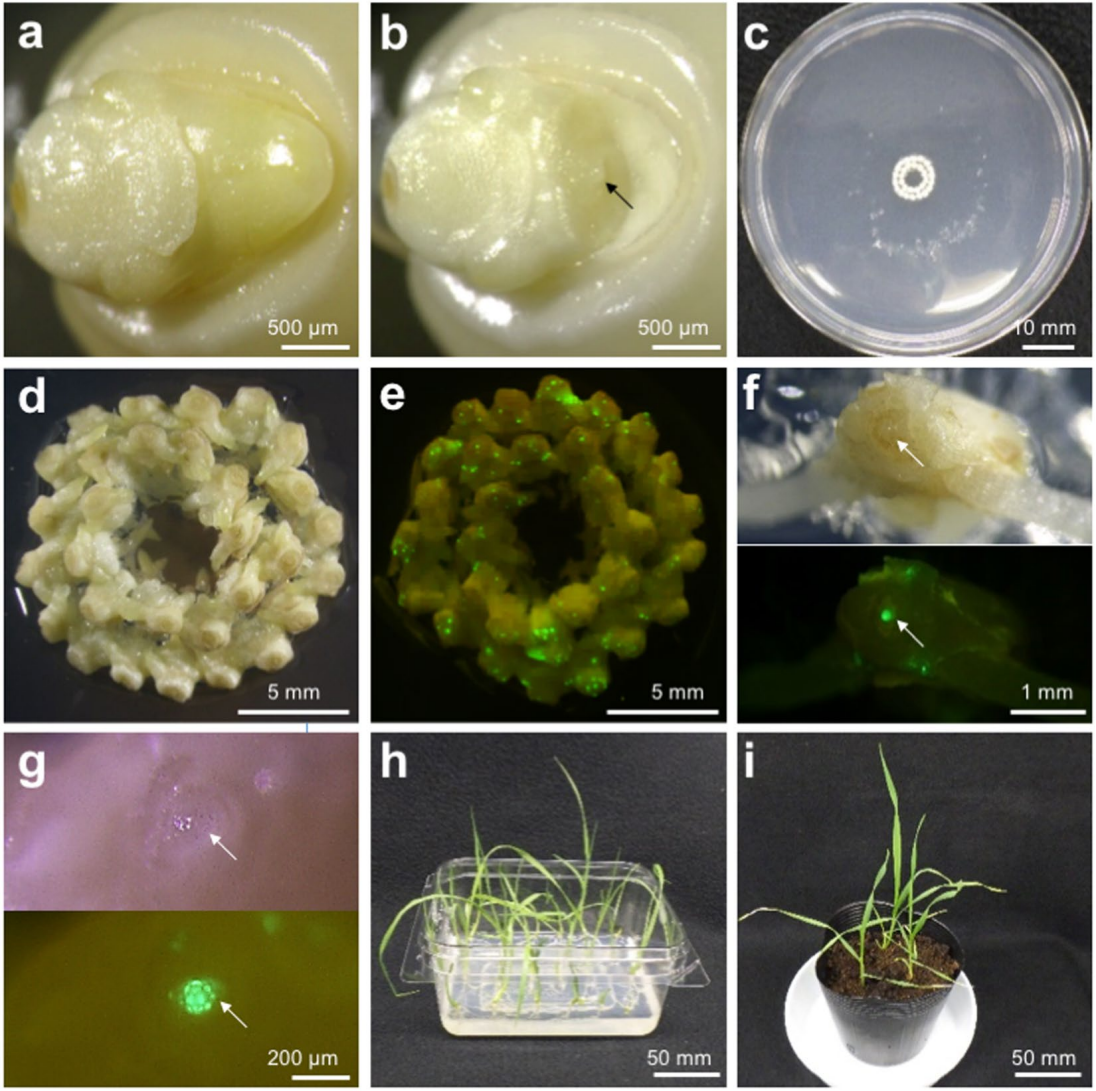
Procedure for *in planta* particle bombardment (iPB) transformation of wheat. (**a**) Coleoptiles and leaf primordia from mature embryos were excised under a microscope, (**b**) and arranged on a culture plate with Murashige and Skoog’s (MS) medium before (**c**) transformation. (**d**) Bright field and (**e**) fluorescence-merged images of the whole apical tissues 12 h after bombardment. (**f**) Bright field (upper) and fluorescence (lower) images of a single apical tissue. (**g**) Close-up images of a SAM region of apical tissue. **(h)** Wheat plants grown on MS medium 3 weeks after bombardment. (**i**) Bombarded wheat plants 2 weeks after transfer to soil. Shoot apical meristems (SAMs) are indicated by arrows in panels (**b**), (**f**) and (**g**).

To establish microprojectile-mediated DNA transfer to the meristematic cells, the *GFP* gene driven by the maize *ubiquitin* promoter (Pubi) was used as a reporter. Coleoptiles and the first three leaf primordia were removed from imbibed ‘Fielder’ seeds to expose shoot apical meristems (SAMs) (Fig. 1a and b). SAM-exposed apical tissues were excised from seeds and arranged in circles on a plate (Fig. 1c). Gold particles of 0.6–1.6 µm in diameter were coated with the reporter plasmid and bombarded into the apical tissue under 1100 psi (7.6 MPa) or 1350 psi (9.3 MPa) helium pressure. After 12 h, the bombarded apical tissues were observed under a fluorescence microscope to check for transient *GFP* expression in the SAM (Fig. 1d and e). GFP signals were detected on the entire surface of the SAM in several bombarded plants; those carrying five or more signal spots were considered GFP-positive (Fig. 1f and g). On the other hand, no wound-induced GFP signal (auto-fluorescence) was observed in the SAM of apical tissues bombarded without GFP plasmid (Supplementary Fig. S1). As shown in Table 1, the percentage of GFP-positive SAMs gradually increased with decreasing gold particle size under 1350 psi (7.5, 35.0, and 74.2%). By contrast, the percentage of GFP-positive SAMs decreased when the particles were accelerated at 1100 psi (21.7, 15.8, and 13.3%). This result suggests that particle size and pressure affects delivery efficiency in a complex manner, but the highest efficiency was observed with 0.6-μm gold particles and 1,350 psi pressure (Table 1).

**Table 1:** Effects of particle size and pressure on gene delivery efficiency.

Particle size (μm)	Helium pressure (psi)	No. of bombarded plants	No. of plants carrying GFP within SAM *(%)	No. of transgenic plants** (%)
1.6	1,100	120	26 (21.7)	0 (0.0)
	1,350		9 (7.5)	0 (0.0)
1.0	1,100	120	19 (15.8)	1 (0.8)
	1,350		42 (35.0)	2 (1.7)
0.6	1,100	120	16 (13.3)	1 (0.8)
	1,350		89 (74.2)	5 (4.2)

*Wheat plants carrying 5 or more GFP signal spots were considered GFP-positive.

**Wheat plants were considered to be transgenic based on positive genomic PCR in the fifth leaf of T_0_ progeny.

Transient GFP expression in SAMs suggested that stable transformation might occur within the meristematic region. Bombarded embryos were grown on basal Murashige and Skoog (MS) medium for 2–3 weeks to allow full root and shoot development, and were then transferred to soil (Fig. 1h and i). Chromosomal integration of the *GFP* gene was tested in the fifth leaf by genomic polymerase chain reaction (PCR) (Table 1). The *GFP* gene was detected when 0.6-µm and 1.0-µm particles were used. No plants bombarded with 1.6-μm particles showed integrated *GFP* (Table 1). With 1350 psi pressure and 0.6-μm particles, the maximum number (5) of transgenic plants were obtained, which correlated with the efficiency of transient GFP expression in SAMs (Table 1). The transgene was detected in subsequently developed leaves (Supplementary Fig. S2). These results suggested that, within the range of conditions tested, 0.6-μm particles and 1350-psi pressure is the optimum combination for transgene delivery into the SAM and the generation of transgenic plants.

### Integration and inheritance of the transgene in transgenic plants

**Figure 2:**
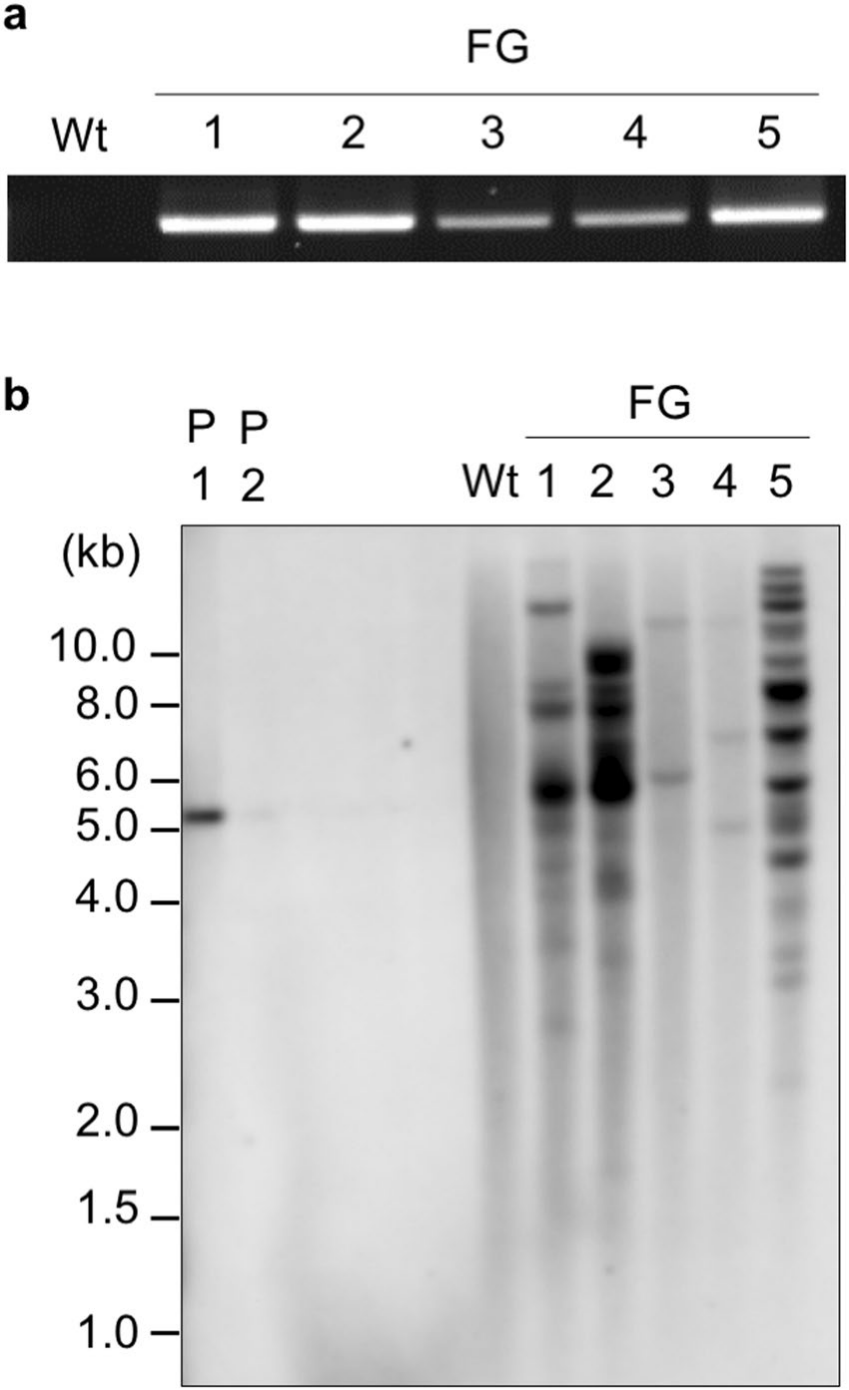
Integration and inheritance of the *GFP* gene in transformed wheat lines. (**a**) Genomic polymerase chain reaction (PCR) analysis of five independent transgenic ‘Fielder’ lines (FG1–5) and wild-type (Wt) lines. Genomic DNA was extracted from each first leaf of T_1_ progeny. The full-length gel image is shown in Supplementary Fig. S6. (**b**) DNA gel blot analysis of transgenic wheat lines (T_2_ progeny) and Wt lines using genomic DNA from leaves digested with *Hind*III. One-hundred picograms (P1) and 10 pg (P2) of linearised GFP vector (5.1 kb) were used as positive controls.

Larger-scale screening of transgenic plants was conducted using the optimal conditions (0.6-μm particles and 1350-psi pressure). Of the 577 bombarded embryos, 379 that transiently expressed GFP in their meristematic regions were selected and grown to adulthood. Eight putative transgenic plants were identified by genomic PCR analysis of flag leaf tissue (Table 2). T_1_ seeds from the primary spike of these plants were subjected to further analysis. Genomic PCR analysis revealed that five (FG1–5) of the eight lines inherited the transgene to the T_1_ generation (Table 2, Supplementary Table S1, Fig. 2a). Existence of *GFP* in the genome was also confirmed by DNA gel blot analysis (Fig. 2b). The number of hybridising bands varied from two (FG3) to over ten (FG5) (Fig. 2b). Chi-square analysis revealed that the FG3 line segregated in a Mendelian fashion (measured value = 50:2, expected value = 15:1, *χ* ^2^ = 0.20, *P* > 0.3). These results suggest that *GFP* was directly introduced into a germline cell within SAMs and was inherited to the next generation.

**Table 2:** Comparison of transformation efficiency between ‘Fielder’ and ‘Haruyokoi’.

Cultivar	No. of bombarded wheats	No. of transgenic plants in T_0_ progeny* (%)	No. of transgenic plants in T_1_ progeny** (%)	No. of T_1_ plants expressing GFP (%)
Fielder	577	8 (1.39)	5 (0.87)	1 (0.17)
Haruyokoi	569	13 (2.28)	4 (0.70)	2 (0.35)

*Wheat plants were considered to be transgenic based on positive genomic PCR in the flag leaf of T_0_ progeny.

**Wheat plants were considered to be transgenic based on positive genomic PCR in T_1_ progeny.

### Expression of the transgene in transgenic plants

**Figure 3:**
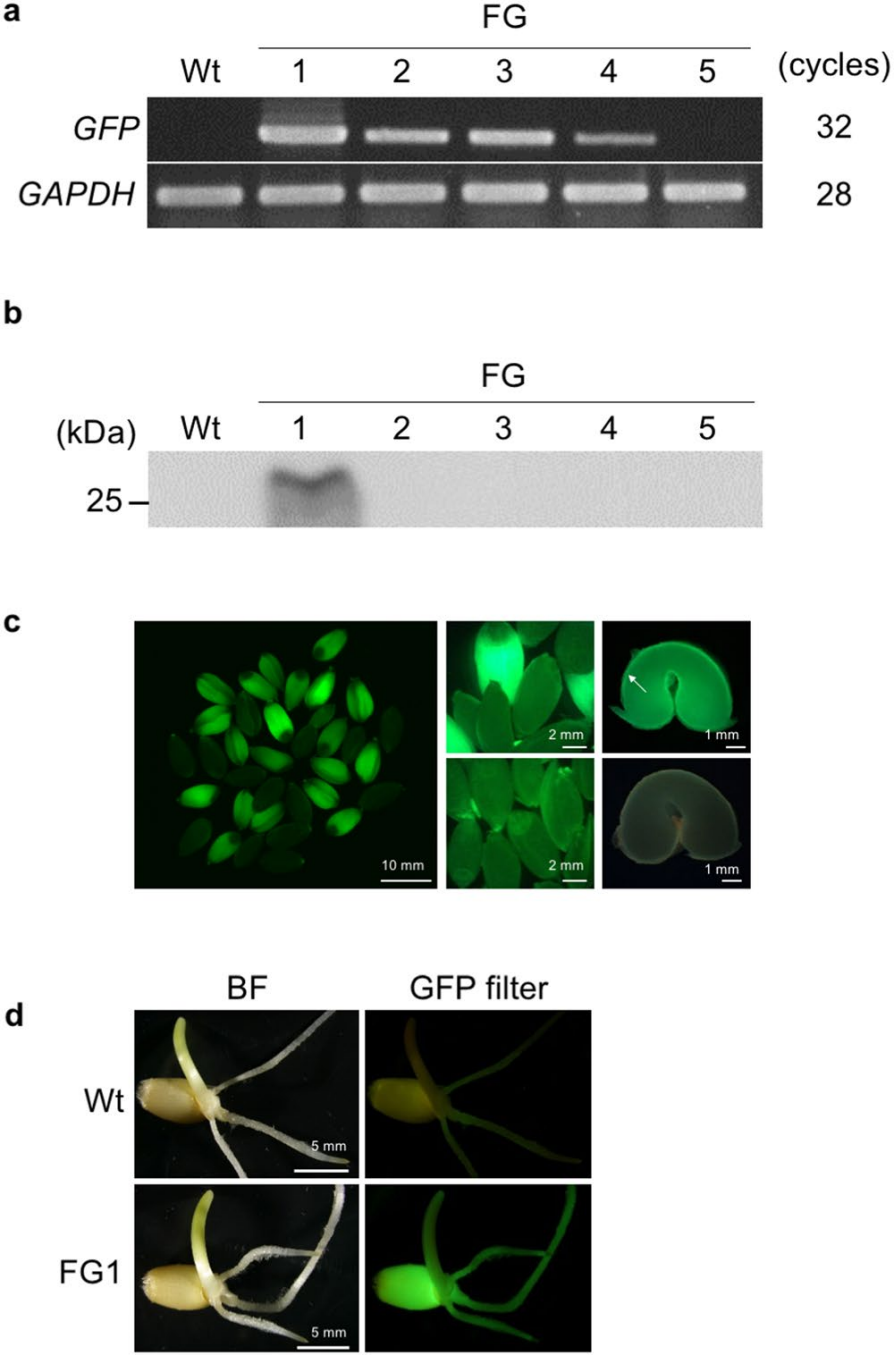
Expression of GFP in transgenic wheat plants. (**a**) Reverse transcription polymerase chain reaction (RT-PCR) analysis of *GFP* in ‘Fielder’ lines (FG1–5) and wild-type (Wt) plants. The *GAPDH* gene (Genbank accession number: EF592180) was used as a housekeeping control. Full-length gel images are shown in Supplementary Fig. S7. (**b**) Immunoblot analysis of green fluorescent protein (GFP) in transgenic plants (FG1–5). Total protein (20 µg per lane) extracted from each T_1_ leaf tissue was separated using sodium dodecyl sulphate-polyacrylamide gel electrophoresis (SDS-PAGE) and blotted. GFP was detected with anti-GFP antibodies. The full-length gel image is shown in Supplementary Fig. S8. (**c**) GFP accumulation in T_1_ seeds of a transgenic wheat line (FG1). Left: fluorescence image of whole T_1_ seeds harvested from the primary panicle of FG1. Middle: close-up views of T_1_ (upper) and Wt (lower) seeds. Right: fluorescence images of sections of FG1 T_1_ (upper) and Wt (lower) seeds. The aleurone cell layer is indicated by an arrow. (**d**) Bright-field (BF, left) and GFP (right) images of T_1_ seedlings in Wt (top) and FG1 (bottom) lines.

We tested *GFP* transgene expression in T_1_ plants by analysing *GFP* mRNA and GFP protein in the transgenic plants by reverse transcription (RT)-PCR and immunoblot analysis, respectively. Both *GFP* mRNA and GFP protein accumulated in FG1, but not in FG5 which shows a complicated transgene integration pattern (Figs 2b and 3a,b). In the other lines (FG2–4), less mRNA accumulated than in FG1, but protein accumulation was undetectable (Fig. 3a and b). Expression of *GFP* was also confirmed by fluorescence detection. In FG1, GFP fluorescence was observed predominantly in the endosperms and aleurone cells of T_1_ seeds, T_1_ seedlings, and in T_1_ leaf stomata (Fig. 3c and d, Supplementary Fig. S3). The particular *GFP* expression pattern in transgenic T_1_ plants was consistent with that of the Pubi-driven *GFP* gene previously reported^16^.

### Genotype analysis in T_1_ plants

To determine whether all T_1_ seed derived from one transgenic T_0_ line had the same genotype, T_1_ seeds derived from spikes of the main shoot and five tillers of FG1 were germinated and their genotypes analysed by genomic PCR and DNA gel blots. Spikes from the main shoot and four of the tillers (all except the third tiller in FG1), contained mostly transgenic T_1_ seeds (Supplementary Table S2). In DNA gel blot analysis of the T_1_ seedlings, hybridisation signals from the spike of the main shoot and tillers were similar (Supplementary Fig. S4). These results suggest that T_1_ seeds produced by a single T_0_ transgenic plant have the same genotype, and one mature-embryo SAM cell is able to differentiate to produce T_1_ seeds on the primary and subsidiary spikes.

### Stable transformation in a commercial wheat cultivar

**Figure 4:**
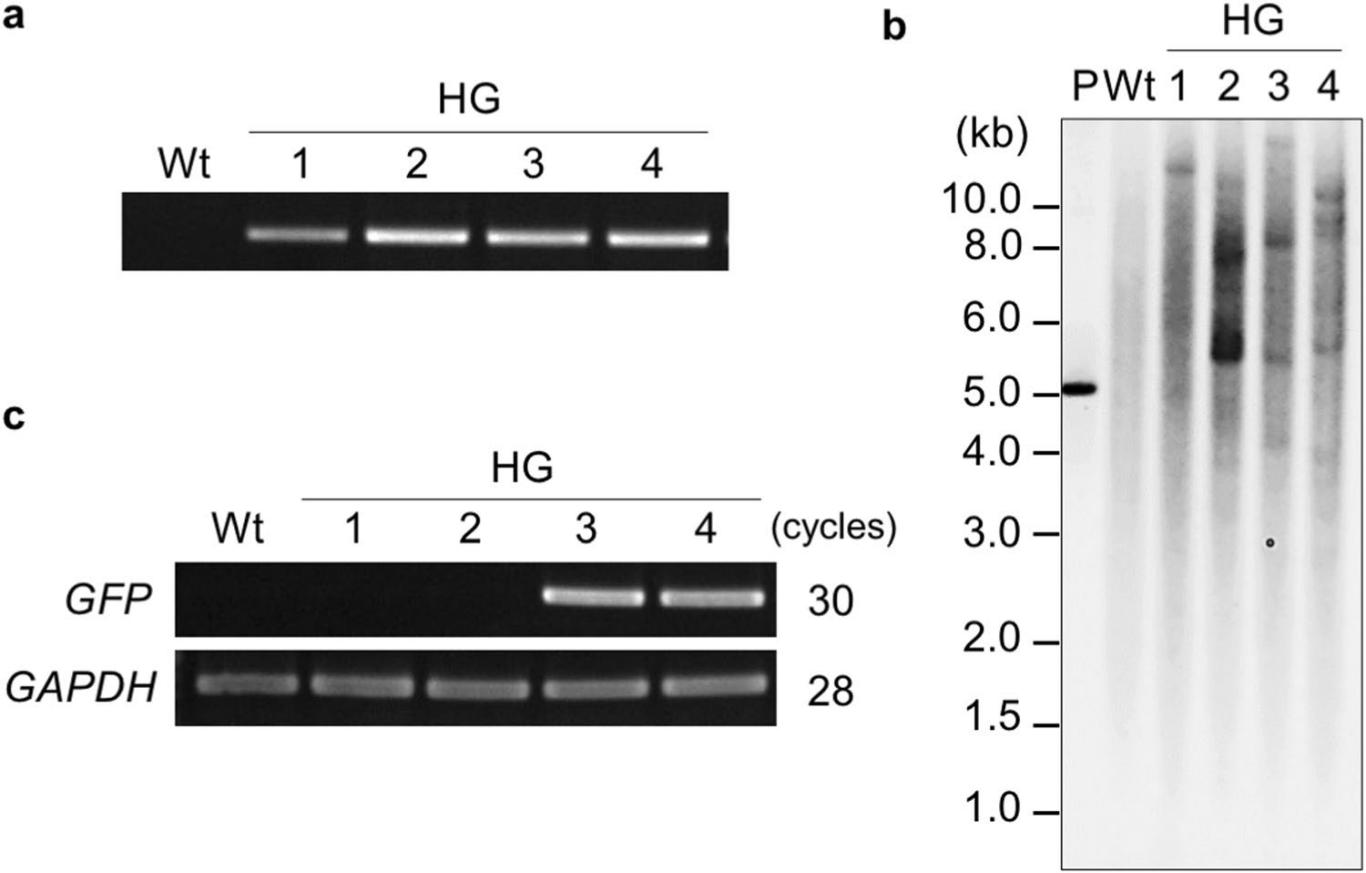
Integration and expression of the *GFP* gene in transgenic ‘Haruyokoi’. (**a**) Genomic polymerase chain reaction (PCR) analysis of four T_1_ transgenic (HG1–4) lines and wild-type (Wt) plants. Genomic DNA was extracted from each first leaf. The full-length gel image is shown in Supplementary Fig. S5. (**b**) Reverse transcription-PCR (RT-PCR) analysis of *GFP* expression in HG1–4 lines and Wt plants. The *GAPDH* gene was used as a housekeeping control. Full-length gel images are shown in Supplementary Fig. S6. (**c**) DNA gel blot analysis of transgenic wheat lines (T_2_ progeny) and Wt lines using genomic DNA from leaves digested with *Hind*III. Two-hundred picograms of the linearised GFP vector (5.1 kb) used as a positive control (P).

Tissue culture and *Agrobacterium*-mediated infection limit the applicability of transformation in many wheat commercial cultivars. Since our method involves no embryogenic callus culture or *Agrobacterium* infection, we applied it to a Japanese elite cultivar ‘Haruyokoi’, whose transformation had never been reported. Of the 569 bombarded embryos, 505 expressed *GFP* in their SAMs. Using genomic PCR analysis of the T_0_ progeny flag leaves, 13 of the 505 plants were selected as putative transgenic plants. Genomic PCR analysis of T_1_ plants showed that four (HG1–4) out of the 13 plants inherited the transgene (Table 2, Fig. 4a, Supplementary Table S1). Integration of *GFP* into their genomes was also confirmed by DNA gel blot analysis (Fig. 4b). Semi-quantitative RT-PCR and GFP fluorescence analyses revealed that two lines (HG3, HG4) expressed *GFP* in T_1_ plants (Fig. 4c, Supplementary Fig. S5). Overall transformation efficiency was comparable to that of the model cultivar ‘Fielder’. These results suggest that the iPB method described here can be used to create transgenic plants in wheat cultivars that are resistant to conventional tissue culture-based methods.

## Discussion

To develop a wheat transformation method that requires no embryogenic callus culture, we applied a biolistic delivery system to apical meristem tissue of imbibed wheat embryos. Our iPB method successfully produced transgenic wheat plants in ‘Haruyokoi’, a leading Japanese cultivar, as well as a model transformation cultivar, ‘Fielder’. Based on transient GFP expression and chromosome integration, transformation efficiency in the T_0_ generation significantly increased when 0.6-µm particles and 1350-psi helium pressure was used (Table 1, Supplementary Fig. S2). We further confirmed that the introduced *GFP* gene was inherited and stably expressed in the T_1_ generation (Table 2, Figs 2a,b and 3a). As demonstrated with ‘Haruyokoi’, since the iPB method requires no embryogenic callus culture or regeneration processes, it can potentially be applied to many commercial cultivars that are difficult to transform via conventional tissue culture-based methods. However, transformation efficiency in the T_1_ generation was approximately 0.8% (Table 2), which is lower than that of the conventional tissue culture-based methods. Further improvement and optimisation is necessary if this method is to be used as a standard protocol.

The use of particle bombardment to directly deliver genes into plant cells is a widely-used technique. However, it can sometimes cause instability in transgene expression. In this study, two patterns of instability were observed. In one of the transgenic ‘Fielder’ lines (FG5), which contains over 10 copies of the transgene, no *GFP* mRNA and or GFP protein was observed (Figs 2b and 3a,b). Transgenes sometimes integrate into a single plant genome locus as a multimer, which may result in gene silencing caused by homologous rearrangements^17,18^. In two other lines, FG3 and FG4, transgene expression was low, although the transgene copy number was also low (Figs 2b and 3a,b). Genomic PCR analysis of the promoter region of *GFP* (Pubi) in the FG3 and FG4 genomes revealed that a ~500-bp segment from the 5′-end of Pubi is missing in these lines (data not shown). This suggests that partial deletion of the promoter might lead to incomplete gene expression (gene repression) in these lines.

SAMs are organised in three cell layers: L1–L3. The L1 and L3 layers are destined to develop into epidermal and vascular cells, respectively. Germ cells such as pollen and egg cells are derived from L2 cells^19,20^. Within the L2 layer, cells that differentiate into male/female gametes are unspecified. Since we observed that at least a portion of transgenic T_0_ plants inherited the transgene into the T_1_ generation, we conclude that particle delivery successfully occurred in the L2 layer cells.

The microprojectile size and acceleration pressure are key controlling factors for particle bombardment. The ratio of SAMs transiently expressing GFP decreased with decreasing particle size in the 1100 psi pressure condition (Table 1). This suggests that a higher velocity might be required for small particles to be able to penetrate the SAM surface. Conversely, when 1,350 psi pressure was used, the ratio increased with decreasing particle size (Table 1). This is likely because the velocity of gold particles accelerated at 1350 psi was sufficient to penetrate the SAM surface and many more plasmids were delivered into SAM cells compared to the other conditions. We also observed that the number of putative T_0_ transgenic plants appeared to increase in line with the above-mentioned ratio (Table 1) and stably transformed transgenic wheat plants were obtained using a combination of 0.6-µm gold particles and 1350 psi helium pressure (Table 2). When 1.6-µm particles and 1350 psi pressure was used, no T_0_ transgenic plants were generated (Table 1). Therefore, we conclude that, within the range of tested conditions, the combination of 0.6-µm particles and 1350 psi acceleration pressure is the best to deliver transgenes into the SAM L2 layer.

In summary, the iPB method described here is able to stably transform wheat without embryogenic callus culture and can be applied to a wide range of wheat cultivars that are difficult to transform with conventional culture-based methods.

## Methods

### Preparation of mature embryos

Mature seeds of wheat (*Triticum aestivum* L.) were sterilised by soaking in sodium hypochlorite (6.0%, 20 min) with a detergent (alkyl ether sulphate), rinsed several times with sterile distilled water, and germinated overnight on moist filter paper at 22 °C. The parts of the coleoptile and leaf primordia covering the SAM were excised with a needle (ϕ 0.2 mm; TERUMO, Japan) under a stereomicroscope. Embryos were subsequently separated from endosperms and placed upright on Murashige and Skoog (MS) medium^21^ supplemented with maltose (30 g L^−1^), 2-morpholinoethanesulfonic acid (MES) monohydrate (0.98 g L^−1^, pH 5.8), plant preservative mixture (3%; Nacalai Tesque, Japan), and phytagel (7.0 g L^−1^; Sigma–Aldrich, USA). Thirty embryos per plate were placed in a circle (diameter 0.8 cm).

### Expression vector

The *sGFP* gene^22^ was cloned into the plasmid pUba^23^ in place of the *bar* gene. The resulting vector (pUba–GFP) allows expression of sGFP under the control of a maize *ubiquitin* gene promoter (Pubi) and the nopaline synthase terminator. Wheat SAMs were transformed with pUba-GFP.

### Preparation of microprojectiles and transformation

Microprojectiles were prepared as previously reported^24^ with slight modifications. Briefly, the plasmid (5 μg) was mixed with gold particles (Bio-Rad, USA) of appropriate diameters in glycerol (60 mg mL^−1^, 50%), spermidine (10 µL, 0.1 M), and CaC1_2_ (25 µL, 2.5 M) in a 1.5-mL tube for 5 min. After incubation at room temperature (10 min), the DNA-coated gold particles were centrifuged (at 9100 × *g* for 2 sec) and the supernatant removed. The pellet was washed with ethanol (70 µL, 70%) and then with 99.5% ethanol. The final pellet was resuspended in ethanol (24 µL, 99.5%) and sonicated (for 1 sec) just before use. Aliquots (6 µL) were spread onto macrocarrier membranes (Bio-Rad, USA) and allowed to evaporate on a clean bench. Bombardment was conducted using a PDS-1000/He™ device (Bio-Rad, USA) with a target distance of 6.0 cm from the stopping plate. The vacuum in the chamber was 27 inches of Hg and the helium pressure was 1100 or 1350 psi. Bombardment was repeated four times.

### Microscopic analyses

SAMs in the bombarded mature embryo and the sections of T_1_ seeds were observed with an MZFLIII microscope equipped with a GFP filter (excitation wavelength, 470/40 nm; emission wavelength, 525/50 nm). Fluorescence images of whole T_1_ seeds and T_1_ seedlings were observed with a fluoroimager LAS3000 (FUJIFILM, Japan) and Olympus SZX16 stereomicroscope equipped with a GFP filter (Ex: BP460–495, Em: BA510IF), respectively.

### Plant growth conditions

Twelve hours after transformation, mature embryos expressing GFP in the SAMs were transferred into a Phytatray™ II (Sigma-Aldrich, USA) with basal MS medium and cultivated for 2–3 weeks in a growth chamber under long day conditions (16 h light/8 h darkness, 22 °C). Seedlings were planted in pots (3 seedlings/pot, ϕ 10.5 cm) and grown in a phytotron under long day conditions (16 h light/8 h darkness, 22 °C).

### Polymerase chain reaction (PCR)

For PCR analysis of the transformed plants, DNA was isolated from the indicated leaf (of the T_0_ progeny) and the first leaf (of the T_1_ and T_2_ progeny), as described previously^25^. PCR amplification to select transformants was performed with Pubi and *GFP* gene-specific primers (Pubi-F, 5′-TTAGCCCTGCCTTCATACGC-3′; and GFP-R, 5′-ACCATGTGATCGCGCTTCT-3′). Each round of PCR was conducted in a reaction mixture (15 µL) containing dNTP and 1 × Ex Taq buffer (0.2 mM of each), primer (300 nM of each), Ex Taq HS polymerase (0.25 U; TaKaRa Bio, Japan), and genomic DNA (about 20 ng). The mixture was denatured (for 3 min at 94 °C) in a thermocycler and then subjected to 30 cycles (T_1_ or T_2_ generation) or 32 cycles (T_0_ generation) of amplification (94 °C for 30 sec, 60 °C for 30 sec, and 72 °C for 30 sec). Half of the individual PCR products was resolved by agarose gel electrophoresis and visualised by staining with ethidium bromide under UV light.

### RT-PCR analysis

Total RNA was isolated from leaf discs using Trizol (Invitrogen, USA) according to the manufacturer’s protocol. First-strand cDNA was synthesised from total RNA (0.5 µg) using ReverTra Ace^®^ qPCR RT Master Mix (Toyobo, Japan). PCR amplification was conducted as follows: initial denaturation (94 °C for 1 min), followed by the indicated number of incubation cycles (94 °C for 30 sec, 60 °C for 30 sec, and 72 °C for 30 sec), and a final extension (at 72 °C for 5 min) using *GFP* gene-specific primers (GFP-F, 5′-ACGGCCACAAGTTCAGCGT-3′; and GFP-R, 5′-ACCATGTGATCGCGCTTCT-3′). *Glyceraldehyde-3-phosphate dehydrogenase* (*GAPDH*) was used as a quantitative control: GAPDH-F, 5′-CAACGCTAGCTGCACCACTAACT-3′ and GAPDH-R, 5′-GACTCCTCCTTGATAGCAGCCTT-3′. Half of the individual PCR products were resolved by agarose gel electrophoresis and visualised by staining with ethidium bromide under UV light.

### DNA gel blot analysis

Genomic DNA (30 µg), extracted by the cetyl trimethylammonium bromide (CTAB) method^26^, was digested with *Hind*III, resolved by agarose gel electrophoresis, and blotted onto a Hybond-N^+^ membrane (GE Healthcare, UK). A *GFP* gene-specific probe was directly labelled with alkaline phosphatase using the AlkPhos Direct labelling kit (GE Healthcare, UK). Hybridisation, washing and chemiluminescent detection with CDP Star were performed as recommended by the supplier (GE Healthcare).

### Immunoblot analysis

Total protein (20 µg) extracted from leaf tissue (1 g) was separated by 12.5% sodium dodecyl sulphate-polyacrylamide gel electrophoresis (SDS-PAGE) and blotted on to a nitrocellulose membrane. The membrane was blocked overnight in 1X TBST buffer (10 mM Tris-HCl, 150 mM NaCl, 0.05% Tween-20, pH 7.5) with fat free milk (5%) at 4 °C. Blots were incubated with anti-GFP mouse IgG1κ antibody (Roche, Switzerland), and then with horseradish peroxidase (HRP)-linked anti-mouse IgG (GE Healthcare). Cross-reacting bands were detected using enhanced chemiluminescence immunoblotting detection reagents (GE Healthcare) and a chemiluminescent analyser, LAS3000 (GE Healthcare).

### Progeny analysis

PCR analysis of leaves of the T_2_ progeny was conducted by detecting the *GFP* transgene. The Chi-square (*χ* ^*2*^) test was used to analyse whether the observed segregation ratio agreed with a Mendelian ratio in the T_2_ progeny. *P* is the probability of the observed ratios reflecting the expected segregation ratio.

## Electronic supplementary material

Supplementary Information
