# Author Correction: An *in planta* biolistic method for stable wheat transformation

**DOI:** 10.1038/s41598-017-17188-2

**Published:** 2017-12-11

**Authors:** Haruyasu Hamada, Qianyan Linghu, Yozo Nagira, Ryuji Miki, Naoaki Taoka, Ryozo Imai

**Affiliations:** 1 Hokkaido Agriculture Research Centre, National Agriculture and Food Research Organization Toyohira-ku, Sapporo, 062-8555 Japan; 2 Biotechnology Development Laboratories, KANEKA CORPORATION, Takasago, 6768688 Japan; 3 Institute of Agrobiological Sciences, National Agriculture and Food Research Organization, 2-1-2 Kannondai, Tsukuba, 305-8602 Japan

*Scientific Reports* **7**:11443; doi:10.1038/s41598-017-11936-0; Article published online 13 September 2017

This article contains an error in Figure 1, where panel D is oriented incorrectly. The correct Figure 1 appears below.

**Figure 1:**
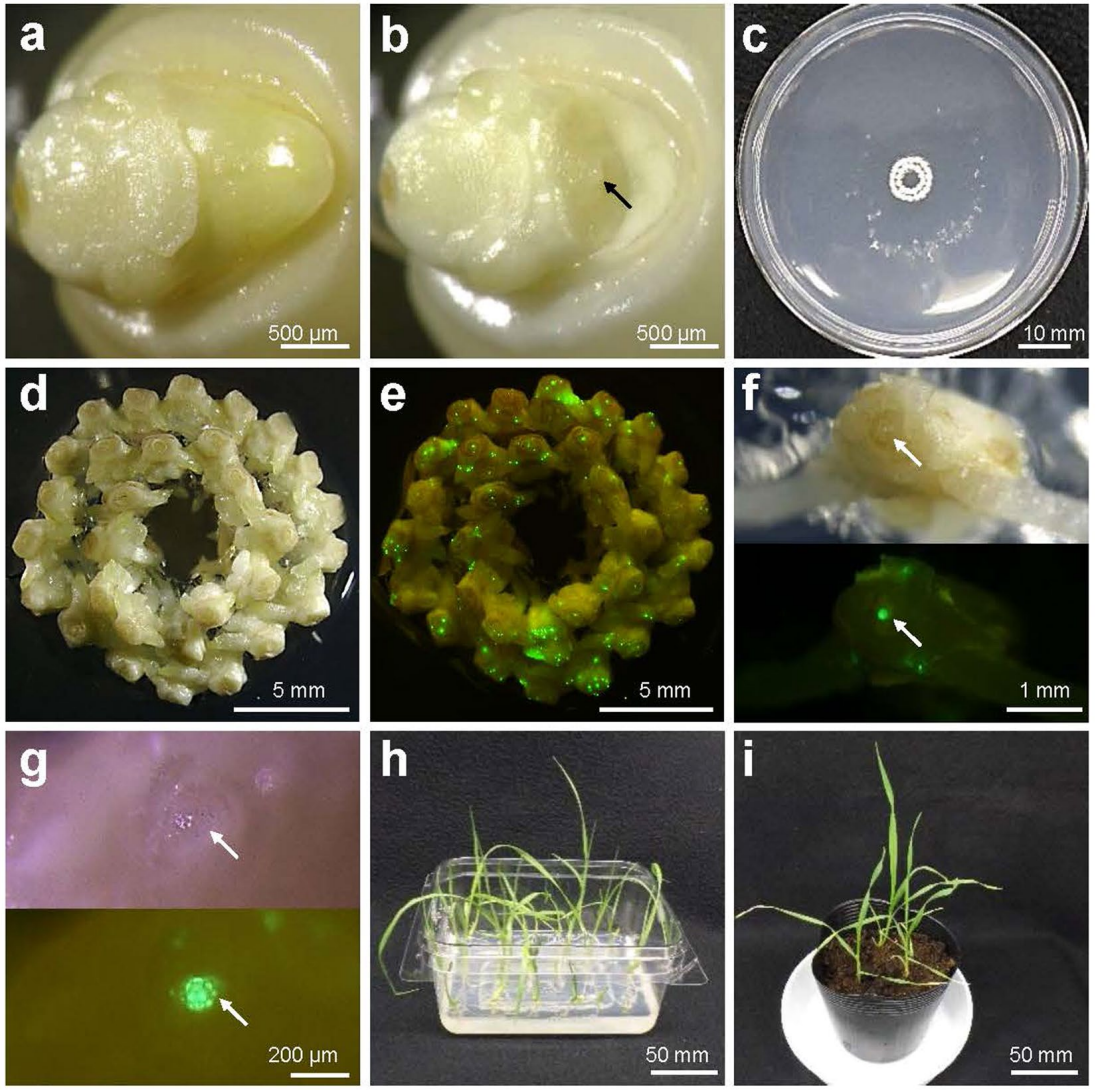
.

