# Supplementary material for: An in planta biolistic method for stable wheat transformation
Source: Sci Rep. 2017 Sep 13;7:11443. doi: 10.1038/s41598-017-11936-0 (PMC5597576; doi:10.1038/s41598-017-11936-0)
Supplement: Supplementary file 1 — Supplementary Information [file 41598_2017_11936_MOESM1_ESM.pdf]

Supplementary Information

An *in planta* biolistic method for stable wheat transformation

Haruyasu Hamada[1,2], Qianyan Linghu [1], Yozo Nagira[2], Ryuji Miki[2], Naoaki Taoka[2] & Ryozo Imai[1,3,*]

[1]Hokkaido Agriculture Research Centre, National Agriculture and Food Research Organization Toyohira-ku, Sapporo 062-8555, Japan.
[2]Biotechnology Development Laboratories, KANEKA CORPORATION, Takasago, Japan.
[3]Institute of Agrobiological Sciences, National Agriculture and Food Research Organization, 2-1-2 Kannondai, Tsukuba 305-8602, Japan.

[*]Correspondence and requests for materials should be addressed to R.I. (e-mail: rzi@affrc.go.jp).

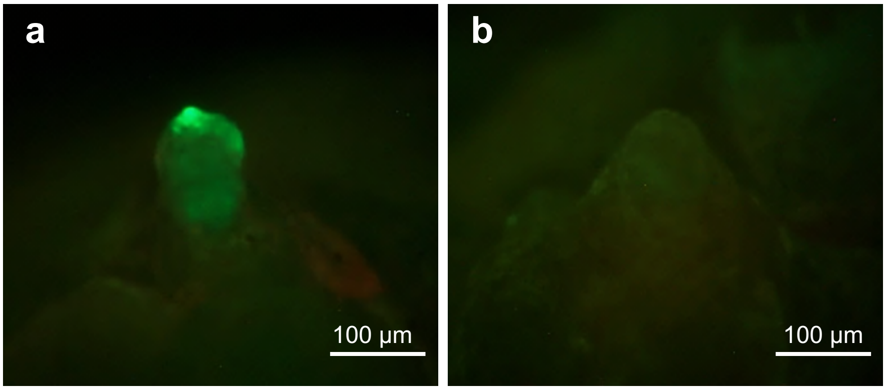

Supplementary Figure S1. Representative GFP images of a SAM bombarded with a GFP expression (a) and empty (b) vectors. Bombarded mature embryos were observed with an MZFLIII microscope equipped with a GFP filter (excitation wavelength, 470/40 nm; emission wavelength, 525/50 nm). The bombardment was carried out with 0.6 µm particles and 1,350 psi helium pressure.

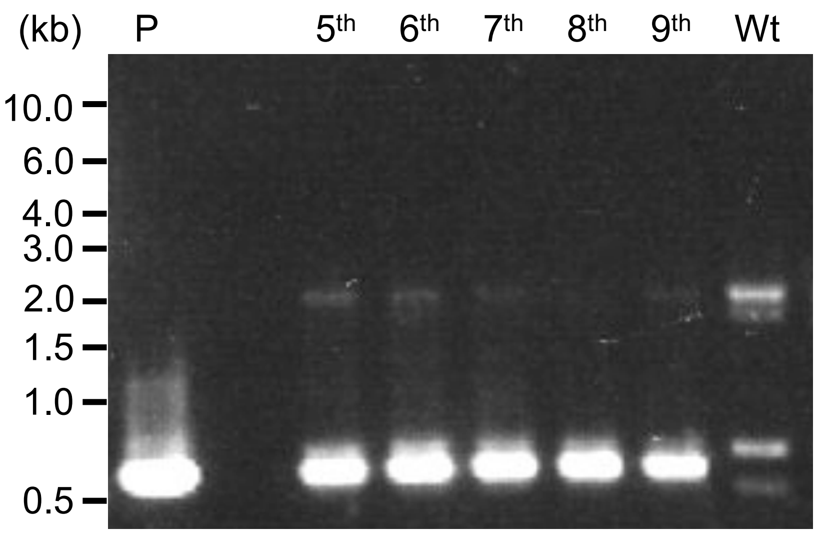

Supplementary Figure S2. Representative PCR screen data from T_0 progeny. Genomic DNA from the indicated number of leaves in a putative transgenic wheat transformed using a combination of 0.6 μm particles and 1,350 psi helium pressure. The introduced GFP plasmid (P, 1 ng) and the fifth leaf from the wild-type (Wt) plant were used as positive and negative controls, respectively.

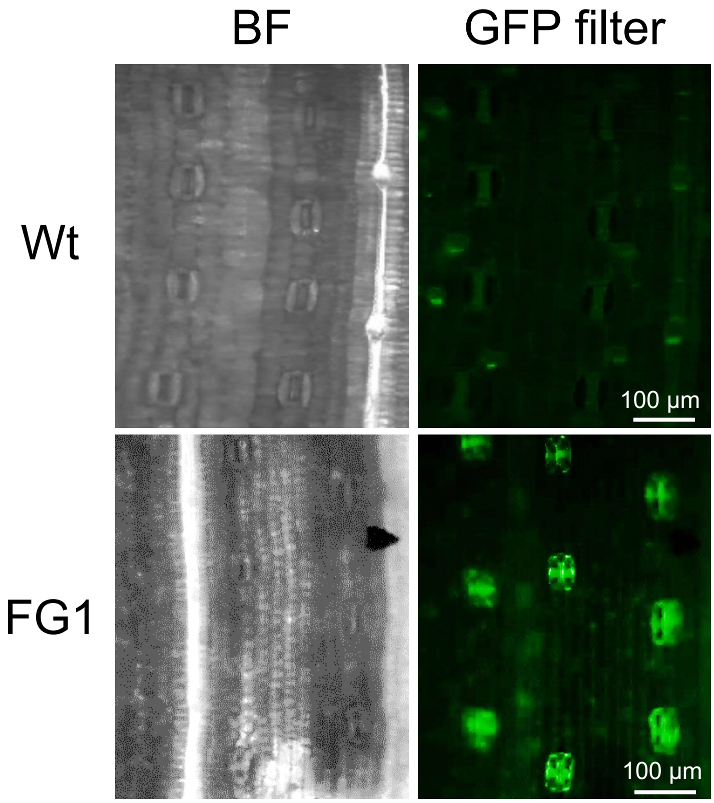

Supplementary Figure S3. Bright-field (BF) and GFP images of T_1 leaf in a transgenic wheat plant. Young leaves of FG1 (T_1 progeny) and wild-type (Wt) plants were sampled and observed under a Leica FW 4000 microscope (Leica, Germany) equipped with a GFP filter (Ex: BP489, Em: 508)

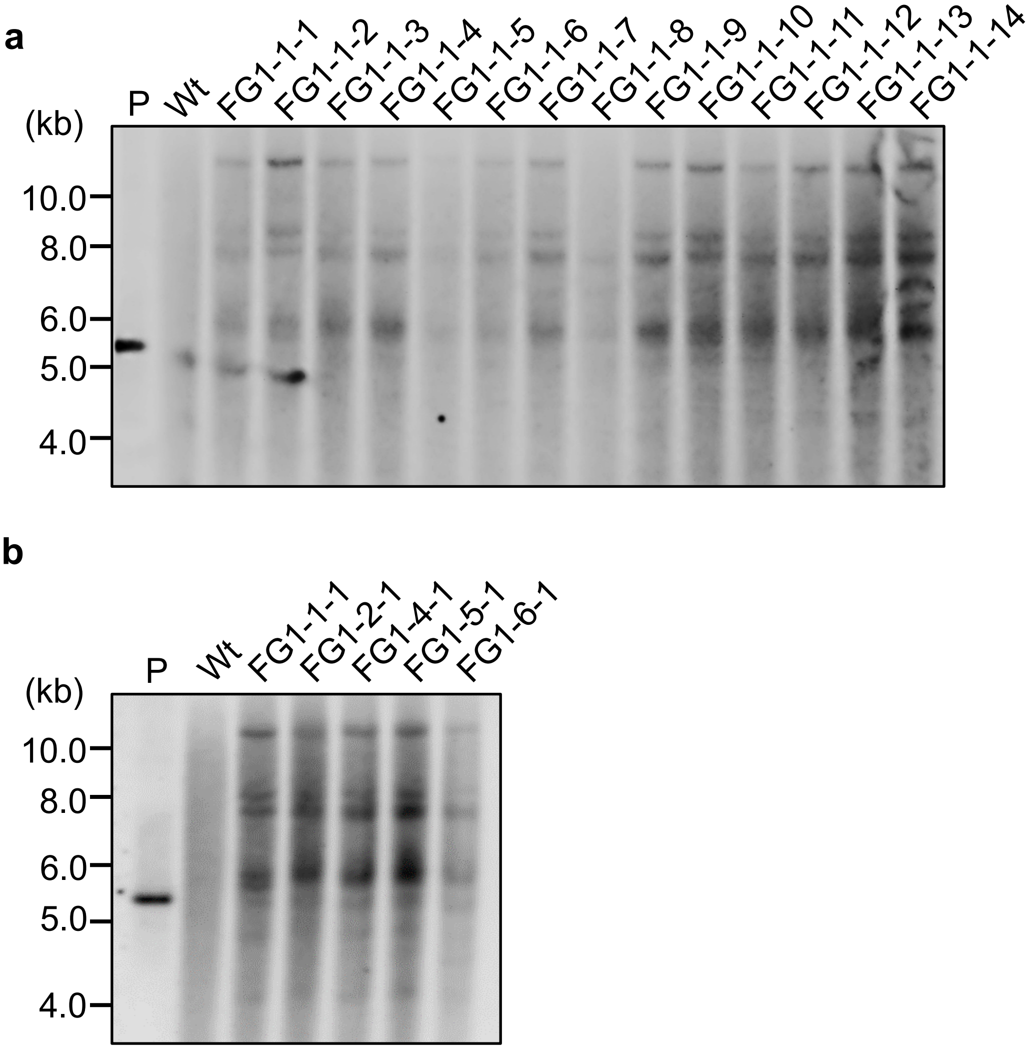

Supplementary Figure S4. Genotypes of T_1 transgenic wheat lines derived from each spike of the T_0 progeny of 'Fielder' line FG1. Transgenic wheat lines carrying *GFP* were analysed by DNA gel blot. Genomic DNA was extracted from each young leaf of the T_1 progeny and digested with *Hind*III. The *GFP* gene-specific probe was hybridised to the blots. **(a)** Lane FG1-1-1~14: DNA from the transgenic T_1 wheat plants harvested from the spikes of the main shoot. Lane P: 200 pg linearised GFP vector (5.1 kb) used as a positive control. **(b)** Lane FG1-1-1: DNA from a wheat plant harvested from the spike of the main shoot. Lane FG1-2-1: DNA from a wheat plant harvested from the spike of 1st tiller, Lane FG1-4-1: DNA from a wheat plant harvested from the spike of the third tiller. Lane FG1-5-1: DNA from a wheat plant harvested from the spike of the fourth tiller. Lane FG1-6-1: DNA from a wheat plant harvested from the spike of the sixth tiller.

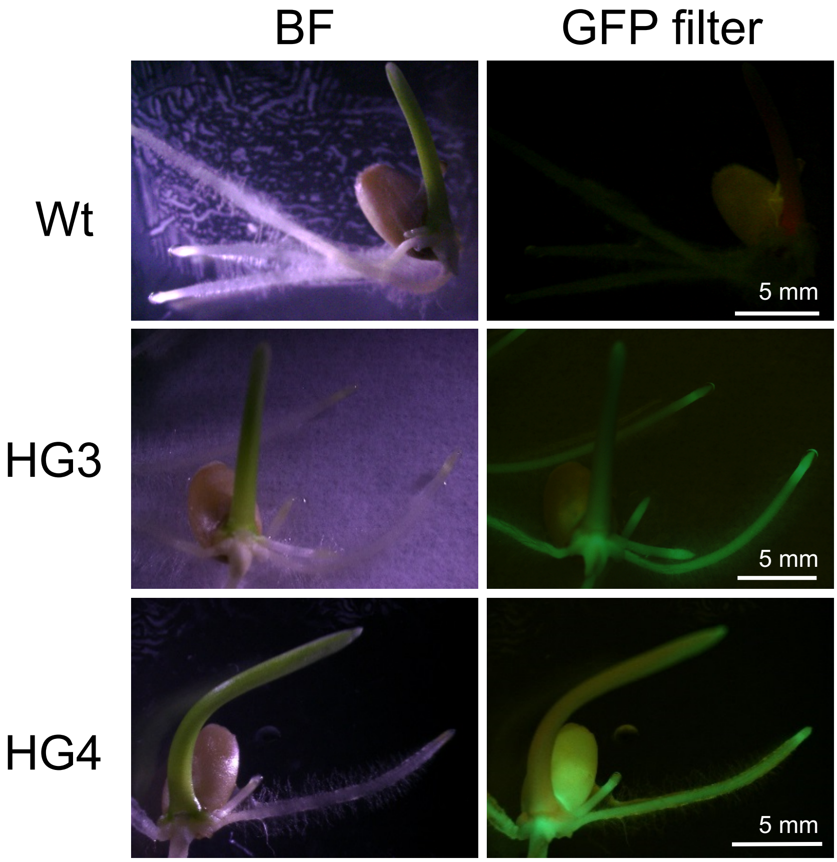

Supplementary Figure S5. Bright-field (BF) and GFP images of T_1 seedlings in transgenic commercial wheat lines. Each T_1 seedling of 'Haruyokoi' lines HG3 and HG4, and wild-type (Wt) was observed under a Olympus SZX16 stereomicroscope equipped with a GFP filter (Ex: BP460-495, Em: BA510IF).

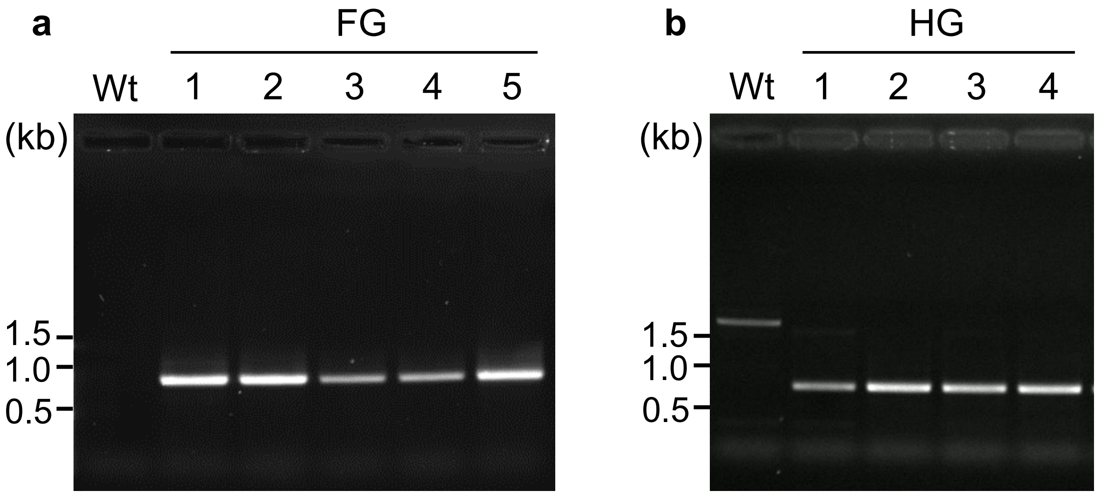

Supplementary Figure S6. Integration of the *GFP* gene in transformed wheat lines. (a) Genomic polymerase chain reaction (PCR) analysis of five independent transgenic 'Fielder' lines (FG1–5) and wild-type (Wt) lines. (b) Genomic PCR analysis of four independent transgenic 'Haruyokoi' lines (HG1–4) and wild-type (Wt) lines. Genomic DNA was extracted from each first leaf. Full-length gel images from Figure 2a and 4a are presented.

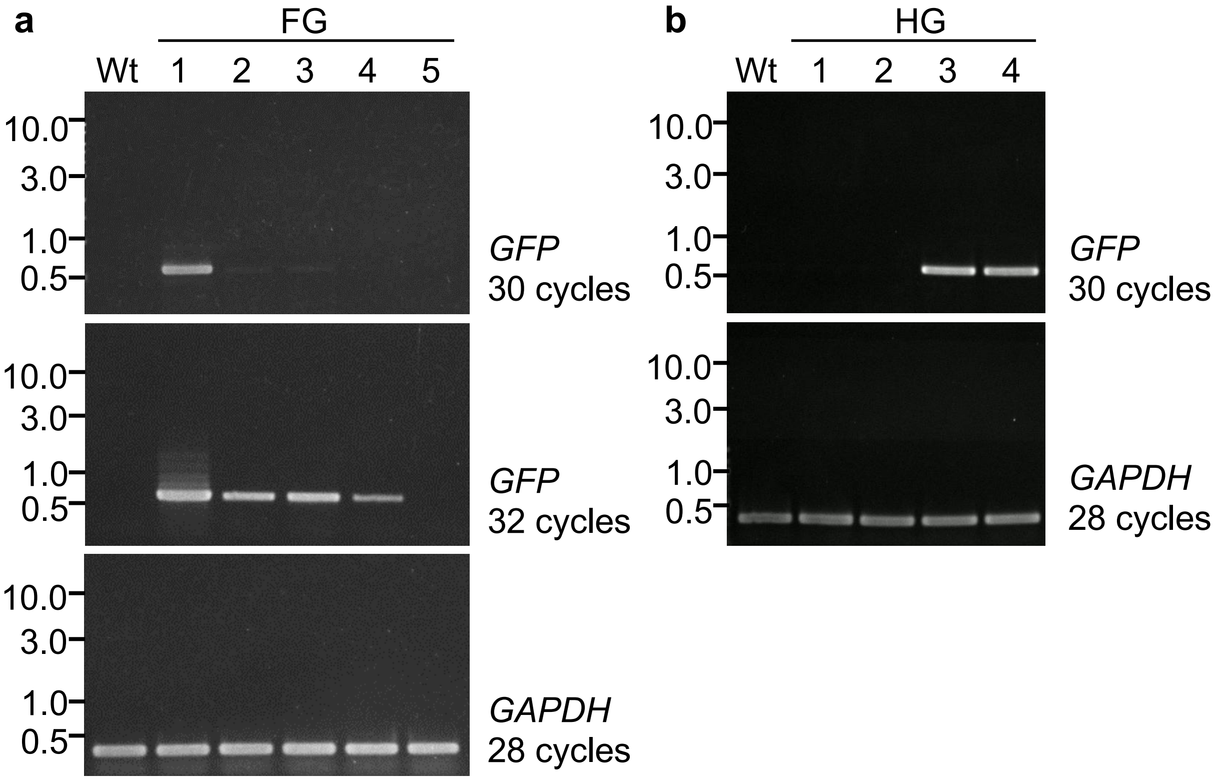

Supplementary Figure S7. Expression of *GFP* in transgenic wheat plants. (a) Reverse transcription polymerase chain reaction (RT-PCR) analysis of *GFP* in 'Fielder' lines (FG1–5) and wild-type (Wt) plants. (b) RT-PCR analysis of *GFP* expression in HG1–4 lines and Wt plants. The GAPDH gene (Genbank accession number: EF592180) was used as a housekeeping control. The full-length gel images from Figure 3a and 4b are presented.

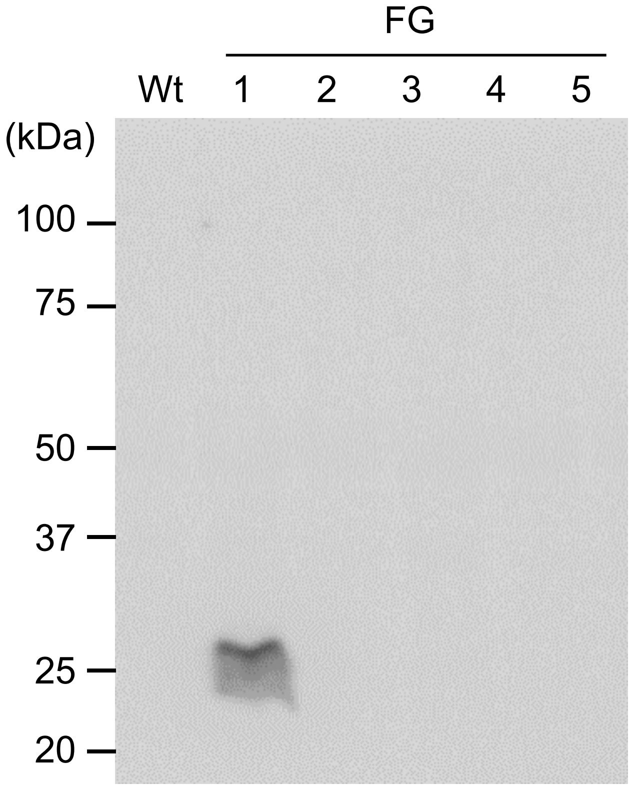

Supplementary Figure S8. Expression of GFP protein in transgenic wheat plants. Immunoblot analysis of green fluorescent protein (GFP) in transgenic plants (FG1-5) and wild-type (Wt). Total protein (20 μg per lane) extracted from each T_1 leaf tissue was separated using sodium dodecyl sulphate-polyacrylamide gel electrophoresis (SDS-PAGE) and blotted. GFP was detected with anti-GFP antibodies. The full-length gel image from Figure 3b is presented.

Supplementary Table 1. Inheritance of the *GFP* gene in T_1 plants

Cultivar	Line ID	No. of T_1 seeds in primary spike	No. of T_1 seeds analysed	No. of transgenic T_1 plants*
Fielder	FG1	35	31	26
	FG2	28	14	10
	FG3	10	10	8
	FG4	48	48	38
	FG5	43	20	1
Haruyokoi	HG1	44	34	5
	HG2	46	36	1
	HG3	48	17	12
	HG4	52	30	22

* Wheat was considered transgenic based on positive genomic PCR in T_1 progeny.

Supplementary Table 2. Polymerase chain reaction (PCR) analysis of T_1 plants harvested from each spike

Spikes	No. of T_1 seeds in each spikes	No. of T_1 seeds analysed	No. of transgenic T_1 plants*
Main	35	31	26
1st tiller	25	24	6
2nd tiller	25	25	0
3rd tiller	27	27	22
4th tiller	23	23	22
5th tiller	7	7	5

* Wheat was considered transgenic based on positive genomic PCR in T_1 progeny.
